# Tails stabilize landing of gliding geckos crashing head-first into tree trunks

**DOI:** 10.1038/s42003-021-02378-6

**Published:** 2021-09-02

**Authors:** Robert Siddall, Greg Byrnes, Robert J. Full, Ardian Jusufi

**Affiliations:** 1 Locomotion in Biorobotic and Somatic Systems Group, Max Planck Institute for Intelligent Systems, Stuttgart, Germany; 2 Department of Biology, Siena College, Loudonville, NY USA; 3 Department of Integrative Biology, University of California at Berkeley, Berkeley, CA USA

**Keywords:** Herpetology, Biomechanics

## Abstract

Animals use diverse solutions to land on vertical surfaces. Here we show the unique landing of the gliding gecko, *Hemidactylus platyurus*. Our high-speed video footage in the Southeast Asian rainforest capturing the first recorded, subcritical, short-range glides revealed that geckos did not markedly decrease velocity prior to impact. Unlike specialized gliders, geckos crashed head-first with the tree trunk at 6.0 ± 0.9 m/s (~140 body lengths per second) followed by an enormous pitchback of their head and torso 103 ± 34° away from the tree trunk anchored by only their hind limbs and tail. A dynamic mathematical model pointed to the utility of tails for the fall arresting response (FAR) upon landing. We tested predictions by measuring foot forces during landing of a soft, robotic physical model with an active tail reflex triggered by forefoot contact. As in wild animals, greater landing success was found for tailed robots. Experiments showed that longer tails with an active tail reflex resulted in the lower adhesive foot forces necessary for stabilizing successful landings, with a tail shortened to 25% requiring over twice the adhesive foot force.

## Abstract (web summary)

Siddall, Byrnes, Full, and Jusufi observe the function of the Asian flat-tailed gecko tail in gliding and landing on tree trunks in the field, complemented with mathematical and robotic models made of soft active materials. Altogether, their models show how geckos use an active tail reflex to brace the impact and reduce the risk of falling from landings on tree trunks.

## Introduction

Geckos are best known for their agile climbing feats using their specialized feet with hairy toes that can uncurl to attach by van der Waal forces and peel in milliseconds^1–3^. As well, active tails play a role in the Asian flat-tailed gecko’s aerial acrobatics^4^. Geckos running at high-speed up a vertical surface encountering a slippery patch initiate a tail reflex in less than 30 msec that pushes their tail into the surface preventing pitch-back. During large slips of its front legs when pitch-back of the head and torso cannot be prevented, geckos avoid falling by placing their tail in an unusual posture similar to a bicycle’s kickstand^4^. Geckos that did fall land right-side-up in a “sky-diving” posture. Further investigation of geckos falling with their backs to the ground shows that a swing of their tail generates a rapid zero-angular momentum, air-righting response in just over 100 msec. No geckos lacking tails could fully perform the maneuver. Geckos’ tails also have been shown to permit controlled swinging of the body under leaves out of sight^5^. Most recently, tails have been implicated in propulsion as Asian flat-tailed geckos race across the water’s surface^6^ at speeds comparable to their speed running on the ground^7^ and climbing up smooth surfaces^2^.

It has been hypothesized^4^ that tails might also have a stabilizing and steering function during gliding. Although gliding and parachuting had been observed anecdotally for a flat-tailed gecko in the field^8^, no behaviors were quantified. More recently, geckos have been placed in a vertical wind tunnel in the laboratory^4^. Despite the fact that these geckos are unspecialized gliders with only modest cutaneous flaps on their body, a flattened tail, and even less prominent webbing between their toes^8,9^, they performed controlled, equilibrium glides (i.e., with a linear trajectory relative to the air, and aerodynamic lift balancing body weight). Moreover, circular tail motion was coupled with turning maneuvers of the body^10^. Gliding geckos that rotated their partially dorsi-flexed tail in a clockwise or counterclockwise direction when viewed head-on initiated turns to the right or left in yaw when viewed from above. As the conservation of angular momentum predicts, when the tail’s motion swung in one direction, the body rotated in the opposite direction producing yaw. Given these laboratory observations, we hypothesized that not only would the Asian flat-tailed gecko, *Hemidactylus platyurus*, execute equilibrium glides in nature, but would likely use their tail to turn and maneuver toward landing sites.

**Fig. 1:**
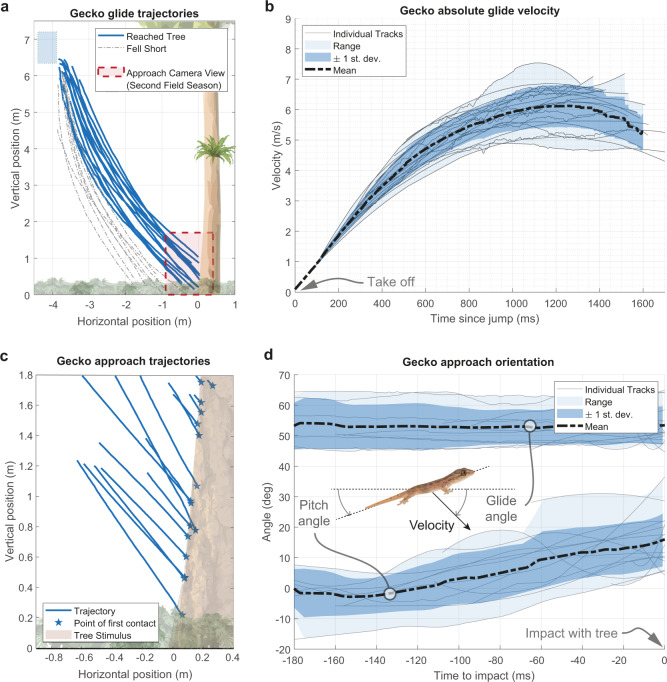
Gecko gliding behavior in short-range, subcritical glides. **a** Trajectories of geckos gliding from an elevated platform to the tree stimulus. A total of 21 trials are depicted, of which 13 reach the tree stimulus (62%). **b** Velocity profile of gecko glides (*n* = 21). 4 out of 21 geckos were still accelerating at impact. Mean velocity decreased 6.4 ± 4.9% relative to peak in geckos that were decelerating (17 out of 21 trials). Glides are aligned in time by linearly extrapolating each run to zero since it was not possible to mark the exact point where the gecko became airborne. **c** Tracked trajectories (*n* = 16) from the approach camera view of gecko landing (Supplementary Movie 1, sequence 2). Note that panels **c** and **d** show different set of trials and individuals from panels **a** and **b**, but using an identical experiment and field site. **d** Glide angle (velocity vector angle) and body pitch angle relative to the horizontal from approach camera view (*n* = 16). Geckos maintained a near constant approach angle, but pitched up their bodies as they traveled, suggesting an attempt to maintain a trajectory by adjusting their body.

**Fig. 2:**
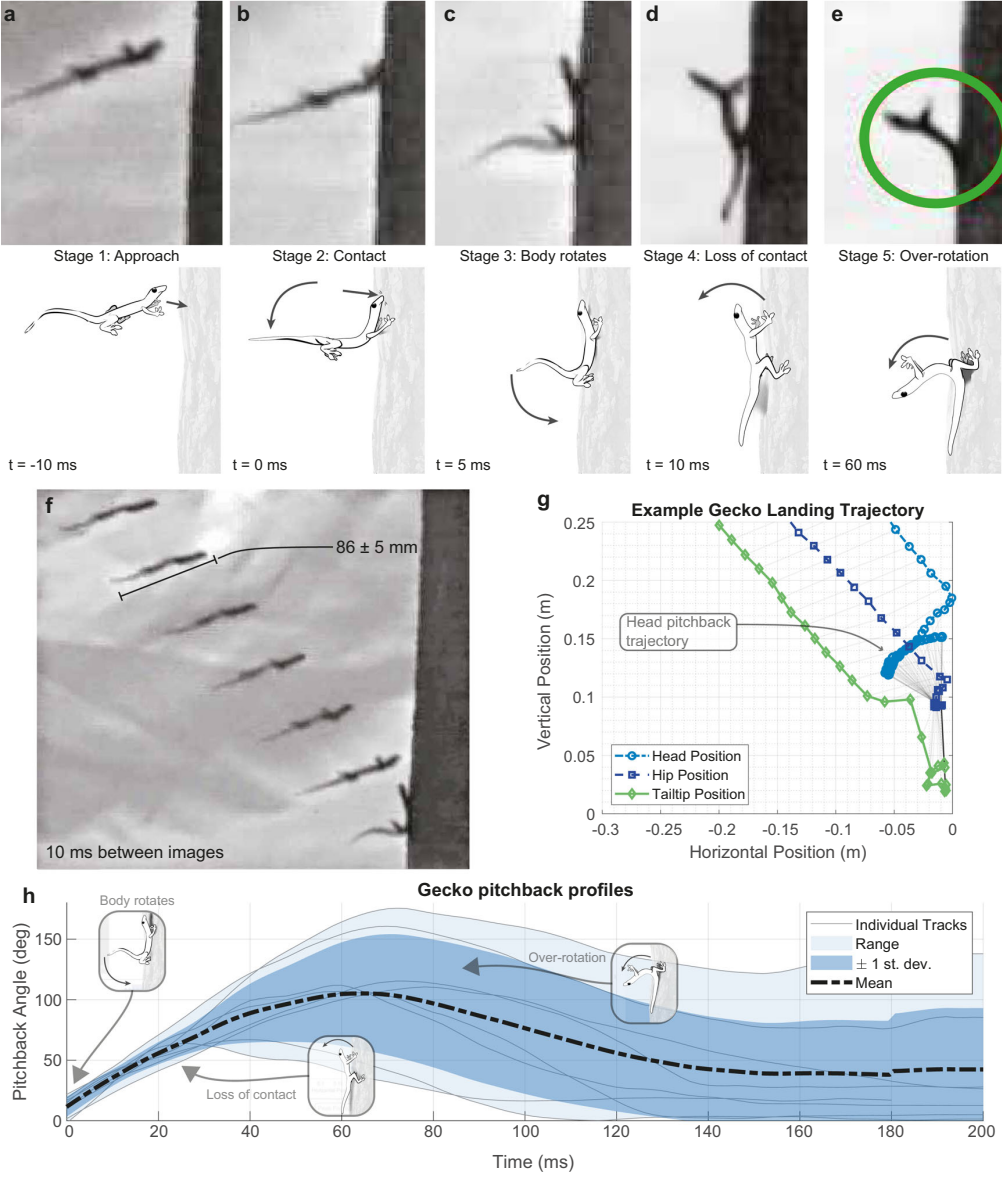
Geckos *H. platyurus* landing on a vertical tree trunk in a Southeast Asian lowland tropical rainforest. (Supplementary Movie 1). **a**–**e** Postural sequence of the landing maneuver and FAR: **a** motion sequence of the gecko approaching the vertical target at positive angle of attack and **b**–**e** alighting onto tree trunk. **b** First impact occurred with the head and anterior trunk. **c** Body rotated downward and rear legs attached to the tree trunk. **d** Front legs slipped off and the gecko’s torso rotated with its back toward the forest floor as the tail made contact with the wall. **e** Maximum pitch-back was attained and the animal momentarily came to rest before its torso returned to the tree trunk so that the front legs regained a foothold. **f** Example approach trajectory, showing an image sequence with 10 ms between stills. **g** Example of body position from landing to pitch-back (*θ*). **h** Body pitch-back during FAR plotted over time (*n* = 8).

Here we present the first quantification, to the best of our knowledge, of the Asian flat-tailed gecko gliding, by capturing high-speed video in the Southeast Asian lowland tropical rainforest. We collected data on glide approach trajectories, glide and pitch angles, and both approach and landing velocities (Fig. 1 and Supplementary Movie 1). We expected that geckos would use a controlled-collision landing strategy as observed in wild, freely behaving flying lizards^11^ (genus *Draco*, several species of which are sympatric with *H. platyurus*). To our surprise, the geckos crashed head-first into tree trunks during subcritical (i.e., with insufficient distance to reach a steady, equilibrium glide), short-range glides^12^ and appeared to stabilize landing using, once again, their remarkably versatile tails (Fig. 2 and Supplementary Movie 1). In fact, we suggest that the tail reflex, along with its bicycle kickstand-like posture observed after slipping during vertical climbing, appears to be conserved when alighting from a glide and used to stabilize landing in nature. We hypothesize that these crash or hard landings are made possible because the tail assists in arresting the substantial pitch-back of the torso by reducing the forces on the hindfeet so that they remain attached to the tree. To test this fall-arresting response (FAR) hypothesis, we complemented our field experiments with two models that examined the effect of tail condition, considering that up to 80% of individuals in lizard populations show evidence of previous tail loss^13^. First, our dynamic mathematical model examines pitch-back with constant angular deceleration, constant tail force, and a proportional tail force to make predictions concerning pitch-back angle and foot forces as a function of relative tail length.

Second, we created a soft robotic physical model that includes a tendon driven tail reflex (Supplementary Movie 2). Robotic physical models can assist in testing biological hypotheses in general^14^, and specifically, we can learn from advances in robotic vertical landings^15–19^, and ultimately enhance robot mobility, enabling perching landing for robotic inspection or quiescence, for example. We launched the robotic physical model at the landing angle observed in geckos toward a vertically oriented force platform (Supplementary Movie 3). We measured landing success, pitch-back trajectory, and landing foot forces for both passive and active tails of varying lengths.

## Results and discussion

Among the suite of aerial locomotion behaviors investigated to date^20–22^, the terminal stage of directed aerial descent or gliding is the area least explored, particularly with respect to absorption of energy on impact in gliders with limited aerodynamic control authority (e.g., without the ability to sustain large angles of attack while steering toward a target). In powered fliers such as birds, bats, and many insects, landing forces can be substantially lower than take-off forces^23–25^. Reduction of kinetic energy before landing or perching is predominantly achieved by flapping wings. Unlike powered fliers, specialized gliders without flapping wings that include flying squirrels, colugos, lizards, snakes, and frogs steer toward landing targets at relatively high speeds and can experience large peak forces at landing while using their limbs, body, or extended skin surfaces^22,25–27^. Flying lizards from the genus *Draco* extend their ribs to attain high glide ratios and can orient their body to be nearly parallel with the surface just before touchdown with all legs simultaneously to slow down and facilitate attachment^11,26,28^. The flying Gecko, *Ptychozoan kuhli*, passively unfurls two large cutaneous flaps laterally between the front and rear legs along with interdigital webbing actively deployed by toe spreading^29^. Experimental restriction of the large lateral flaps or feet has shown that both enhance glide performance^29,30^. Despite its capability of dorsoventral flattening and elongated ribs, sawtail lizards (*Holaspis guentheri*) instead take advantage of low wing loadings via reduced skeletal density to produce their descent trajectories^31^.

By contrast, we estimate that the Asian flat-tail geckos studied here have very high wing loading (~65 N/m^2^) compared to most gliders, even those of greater body mass (see Fig. 3 in Socha et al.^22^). By recording glide trajectories, we found that their glide angle was nearly twice as steep and glide ratio nearly half that shown in specialized gliders such as *Draco* lizards. As a result, Asian flat-tail geckos exhibited short-range, ballistic, unsteady glides with high-speed, high-impact landings (Fig. 1 and Supplementary Movie 1). Typically, geckos were accelerating over the entirety of their glide, and only reached equilibrium near the end of their flight, if at all (4/21 geckos were still accelerating at impact, Fig. 1b). In over half of our trials, gliding geckos reached their target, a tree in a small clearing (13/21 trials with 16 individuals, Fig. 1a). The geckos’ landings on the tree were discernible in 7 trials out of 13 glides that reached the tree, of which one intact gecko and two tailless geckos were unable to withstand landing (Fig. 1a).

**Fig. 3:**
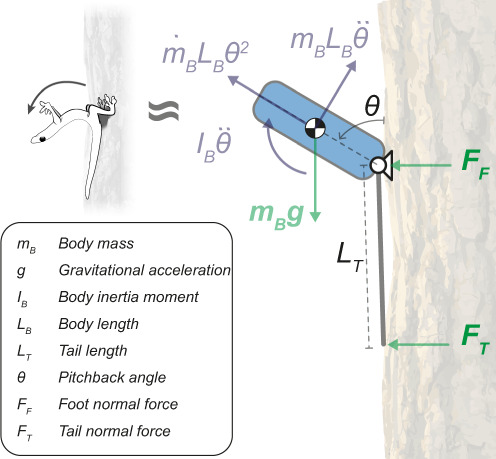
Free body diagram depicts forces acting on the system during the peak excursion phase of the fall-arresting response. Body position *θ* corresponds to the pitch-back angle that is enclosed by the geckos’ torso and the tree trunk. External forces are indicated in green, bold font, whereas virtual forces are shown in violet.

### Short-range gliding approach with limited aerodynamic control

The gliding capabilities of the relatively unspecialized Asian flat-tail gecko resulted in ballistic, short-ranged dives with head-first crash landing velocities of 6.0 ± 0.9 m/s (Fig. 1 and Supplementary Movie 1). *H. platyurus* exhibited postural changes during gliding, and pitched up gradually as they neared the target, but maintained a constant approach angle of 53 ± 5.8° (Fig. 1d), similar to more specialized gliding geckos (*Ptychozoon kuhli*: 52°^29^, *P. lionatum*: 57°^30^). This suggests a behavioral repertoire with limited ability to perform directed aerial descent using available aerodynamic control authority to change posture while maintaining an approach vector. By plotting the velocity over the glide duration (Fig. 1b), we also see that as they approached the tree target geckos reduced velocity, suggesting an attempt at a dedicated landing maneuver. However, lacking specialized aerodynamic morphology, 4 out of 21 geckos were still accelerating at impact, and the animals which decelerated were only able to reduce their speed by 6.4 ± 4.9% from their peak flight velocity, and must absorb almost their full flight momentum on impact. Mammalian gliders can reduce velocity up to 60% prior to landing^27^. Landing at high speed with minimal means of aerodynamic control could increase the risk of injury or falling to the forest floor^32^ with increased exposure to predation. Interestingly, some larger gecko species even appear to have morphological specializations of the skull to reduce head injury risk^33^. Jayaram et al.^34^ compared the specific energy of animal head-on collisions with the maximum energy absorbed by skin and bone. They found that energy dissipation shows a substantial advantage at the small size studied here, permitting mechanically mediated transitions without damage.

### Stages of landing stabilization for fall-arresting response

Original footage from the field in the lowland tropical rainforest revealed that gliding, Asian flat-tailed geckos use their tails in conjunction with their rear legs to land safely on a tree trunk (see Fig. 2a–f). In short, the head-first impact imparts a large amount of pitching angular momentum to the animal. By using their tails to create a long moment arm, geckos are able to gradually dissipate this momentum by pitching back, and ultimately alight successfully with reduced forces. We have termed this remarkable maneuver the “fall arrest response” (FAR). In addition to the 21 glides recorded at long range, we recorded 16 trials of geckos landing on the tree with a close-range camera (Fig. 1c, d), with 5 able to maintain grip with all four feet during landing. One tailed individual fell from the tree (in 1 out of 16 trials), pitching backward but losing grip with hindfeet, and falling to the forest floor. In 2 out of 16 trials, the gecko landed successfully but the landing maneuver was not quantifiable. When tailed animals lost grip with the front feet while crashing into the tree, they exhibited the kickstand-like FAR with pitch-back and landed successfully every time (in 8 out of 16 trials). By contrast, we observed tailless geckos attempting to land upon reaching the tree, losing contact and falling (2 trials). The tailless animal’s loss of front feet contact (Supplementary Movie 1) suggested that the tail could support the stabilization of body pitch-back (Fig. 2d, e). The remarkable landing behavior was executed successfully after tailed animals collided with the tree trunk at high-impact speeds over 6 m/s. A possible explanation for the use of the tails is that the limits of foot adhesive forces required to maintain contact would otherwise be exceeded as has been suggested for some substrates^35,36^.

The animal’s landing maneuver can be characterized by five stages. First, gliding geckos reached the target with their body pitched upward by only 16 ± 8.4° from the horizontal (Fig. 2g). As the animal concluded its aerial descent (Stage 1, Fig. 2a), initial contact occurred with its head and anterior portion of the torso absorbing kinetic energy (Stage 2, Fig. 2b). The collision increased angular momentum that produced a downward rotation of the torso toward the tree trunk. Next, the rear legs contacted the vertical substrate (Stage 3, Fig. 2c). With all four feet on the vertical target, the animal did not slide down the tree toward the forest floor, but instead arched its distal tail tip in the ventral direction. In the fourth stage, both of the forefeet begin to exceed attachment limits and lose contact with the tree (Stage 4, Fig. 2d). In the final stage, pitch-back with the tail pressed against the tree could not be prevented, and the animal’s torso began to rotate backward away from the tree (Stage 5, Fig. 2e). Loss of contact and pitch-back was observed in 8 trials with 7 individuals. Body angles of up to 175° (mean 114 ± 16° s.e.) were recorded (Fig. 2h) when the landing position on the convex tree trunk allowed measurement of body angle. The most spectacular landing pose observed is illustrated in Fig. 2e. This surprisingly large pitch-back response is nearly double the amount that we observed in the “kickstand response” after slipping while rapid wall running^4^, but does support the possibility of a conserved tail reflex used for a different behavior. In wall running^4^, the time between front foot slip and contact from tail reflex was 47 ms. In the FAR, the time taken between loss of front foot contact and peak pitch-back was approximately 64 ms (Fig. 2h), indicating enough time for the reflex to act, despite the increased movement speeds involved in gliding. It is likely that pitch-back in FAR allows more gradual dissipation of energy and is critical to a successful landing. The torso’s angular position (*θ*) as a function of time throughout the FAR is depicted in Fig. 2h. Geckos exhibited substantial pitch-back of their torso away from the tree at a rate of 2057 ± 762°/s to an average angle of 114 ± 16° toward the forest floor (*n* = 8). We measured a mean duration of 138 ± 15 msec for completing the FAR from the gecko’s trunk becoming dislodged to regaining tree contact with front legs (Supplementary Movie 1). In the fifth stage, the gecko recovered by pitching its torso forward toward the tree trunk regaining forefoot contact (Fig. 2h).

### Dynamic model of fall-arresting response

To gain insight into the possible effect of tails in determining landing success, we developed a simplified, planar rigid body dynamics model of the gecko during pitch-back of the FAR. From the video footage taken in the rainforest, we observed that the geckos’ hind legs do not regress or slip during the pitch-back maneuver. To quantify the FAR, we represented the hindfeet as a pin joint, which exhibited a reaction force component normal and tangential to the vertical tree surface (Fig. 3), and the gecko’s torso by a single rigid uniform body rotating about that joint (the blue oval in Fig. 3), with length ${L}_{B}$.

Compared with the gecko adhesive system of the foot gripping the tree, the tail friction that the smooth, large, and unspecialized subcaudal scales can generate is orders of magnitude lower. In addition, the tail friction can only contribute minimal pitching torque to the body, owing to the small moment arm between it and the pivot point at the hindfeet. Therefore, we treat it as negligible for the outcome of this behavior. We propose that the system can be further simplified by assuming negligible friction forces from the tail and the tree, as well as a ridge transfer of forces through the spinal vertebrae and the axial and hypaxial musculature, such that the tail force, ${F}_{T}$, can be represented as a point load normal to the vertical tree surface, located at a distance, ${L}_{T}$, from the pin joint, through which forces can be rigidly transmitted to the torso. A full derivation of the resulting equations of motion is found in the Methods section.

### Tails reduce foot forces and falling

**Fig. 4:**
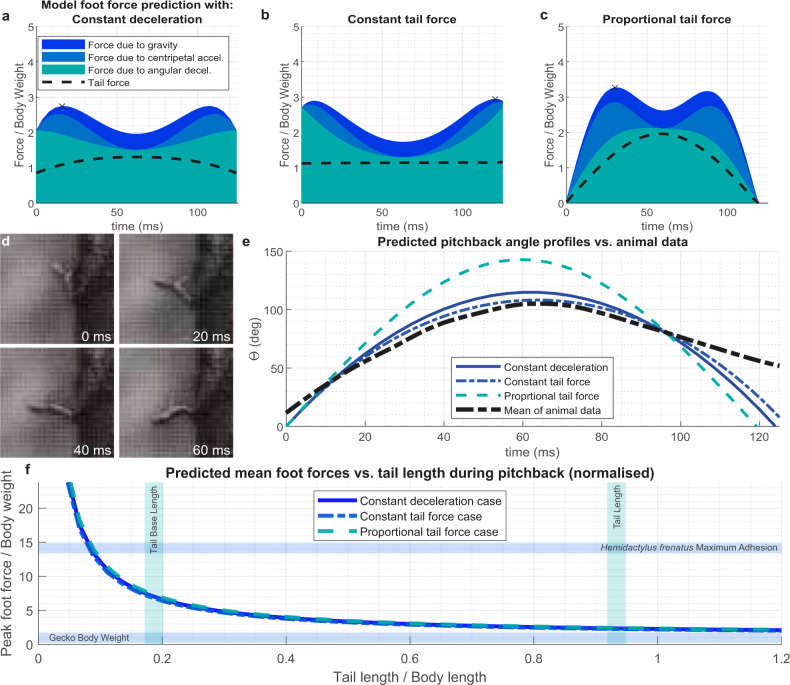
Dynamic model predictions of fall arrest response. **a**–**c** Modeled foot force profiles during FAR, with constant angular deceleration, constant tail force, and a proportional tail force, respectively. **d** Gecko pitch-back image sequence. The initial angular velocity measured in the wild gecko was used as a boundary condition for the model solution. **e** Comparison of model and animal pitch-back time history. The constant angular acceleration case shows a similar maximum pitch-back angle and pitch-back duration as in the animal data. **f** Peak foot adhesion force required versus tail length, as predicted by the model. Shorter tails require higher foot adhesion forces, which could explain the lower success of tailless animals. By solving the model equations for a range of tail lengths, we observe the relationship between tail length and landing adhesive force. We found consistency between the three simple solution cases tried (see Fig. 4a–c) showing that a shortened tail results in greatly increased foot forces. On the plot, vertical lines indicate the tail length and the length of the tail base in caudotomized geckos. Horizontal lines indicate the mean body mass of the captured geckos and a measured adhesion force on glass from a closely related species of gecko, *Hemidactylus frenatus*.

Given our dynamic model estimates, the rear foot force, ${F}_{F}$, required to keep the lizard attached to the vertical substrate would be inversely proportional to tail length (Eq. 4, Methods). While this indicates the importance of tails, it is does not demonstrate that the maximum pitch-back condition is the critical point at which detachment of the hindfeet would result in a fall. To explore foot force conditions over the entire pitch-back maneuver, we can instead examine three cases for which simple numerical solutions to the equations of motion are possible (see Methods section for calculation details). These three cases are constant angular deceleration ($\ddot{\theta }$ = constant), constant tail force (${F}_{T}$ = constant), and a proportional tail force (${F}_{T}$/ $\theta$ = constant), where the constant in each case can be used as a fitting parameter. Physically, constant deceleration would represent the minimum torque at the pivot point and the most gradual deceleration of the pitch-back. A constant tail force would represent the maximum mechanical work that could be applied if tail force was limited by the tail musculature. A proportional tail force would represent an elastic response from the body tissue. In reality, the true tail response is likely to be a combination of these cases, e.g., tissue elasticity and active muscular force combined. For each, we predict pitch-back forces for the observed geckos using the initial pitch-back angular velocity measured during the mean pitch-back (Fig. 2h) as an initial condition, and set the mass and length of the rigid body in the model to the mean body mass and length of the animals (see Supplementary Table S1). The results from this analysis are illustrated in Fig. 4a–c. It is apparent that the largest component of force in each case is due to the angular deceleration of the body, which represents the gecko dissipating the kinetic energy accumulated during its glide. By comparing predicted pitch-back angle profiles with the measurements of the actual gecko (Fig. 4d, e), we see concordance in both peak pitch-back angle and duration in the constant deceleration case. Constant deceleration would require minimum force over a given deceleration period.

We can extend this analysis and predict the mean tail force across a range of values of tail lengths (Fig. 4f). We find the inverse proportionality relationship indicated by Eq. (4) in the Methods remained dominant even when the other terms in Eq. (3) are included (see Fig. 10 in^15^). By comparing the typical length of the tail base left after caudal autonomy in a gecko, we can see that this length represents a sudden and steep increase in the force requirements in the foot to levels an order of magnitude above body weight, or greater.

The model predictions are consistent with our findings of reduced tailless animal performance. This implies that animals with long tails can effectively reduce ${F}_{F}$ required to keep the gecko attached to the tree by its rear legs as it counteracts large overturning moments. By contrast, tailless lizards’ ${L}_{T}$ is much shorter than in lizards with intact tails. Thus, it follows that larger foot forces are required to ensure a successful landing in tailless geckos, potentially exceeding the critical strain level that the rear legs can sustain. According to this prediction, the specific consequence for *H. platyurus* is that foot forces required to keep a tailless gecko attempting to land from falling off would be approximately five times greater than those for tailed geckos (Fig. 4f). While we do not know the critical ${F}_{F}$ or the maximum force with which the rear legs would be able to cling to the tree before becoming dislodged, it does appear that for a given landing on vertical substrate the ${F}_{F}$ in tailless animals are more likely to exceed this threshold, leading to a fall. This provides a mechanistic explanation for the function of the tail in stabilizing the landing maneuver. Moreover, it is consistent with the performance difference observed in the field where successful landings were found in the vast majority of trials with tailed geckos that glided to the tree (87%, *n* = 23, combining observations from 16 trials with the close-range camera angle, and 7 trials from the long range camera angle in which the landing is visible) compared to catastrophic falling observed in the tailless animals that lost stability upon collision, fell head over heels, bounced off in an uncontrolled fashion to fall onto the forest floor (Supplementary Movie 1). Experiments with geckos being confronted with perturbations while running vertically revealed that they can push into the wall to counter pitch-back induced by foot slippage, whereas tailless animals fall head over heels^4^. Geckos with intact tails were also able to use the larger moment arm to effectively overcome even larger slippery gaps as they exhibited the “kickstand response”^4^ that allowed recovery from substantial pitch-back angles of up to 60°. A similar mechanism is likely used to control landing.

These results support the hypothesis based on field observations that tails are used to stabilize crash or hard landings and control high-impact forces acting on the limbs, allowing effective perching on vertical targets at high speed. To provide an independent line of evidence for this hypothesis to complement the mathematical model, we have also conducted experiments with a scaled robotic physical model that includes collection of force data.

### Robotic physical model supports landing stabilization by tails

Studying the perching dynamics using a physical model^15–17^ allows direct measurements of the estimates from our mathematical model. We used a dynamically similar robotic model to test the stabilizing effect of tails on perching robustness. We measured the forces generated by launching a robot model onto a vertical landing surface using a catapult at prescribed speeds between 3 and 5 m/s (see Supplementary information, Supplementary Fig. S1) at the approach angles measured for geckos in the field (Fig. 1c, d). Details of the robot sizing, construction, experimental setup, and measurement procedure can be found in the Methods section.

### Physical model kinematics

**Fig. 5:**
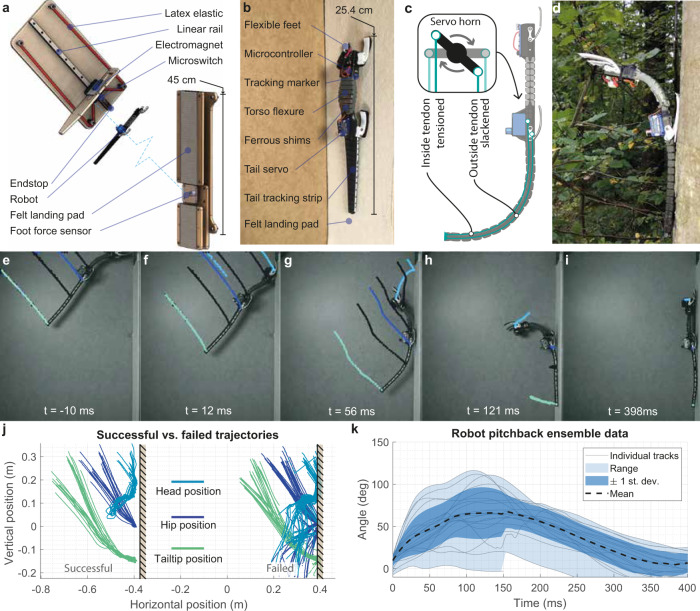
Landing pitch-back behavior in robotic physical model with tail. (Supplementary Movie 2). **a** Rendering of catapult and landing pad setup, showing robot model and force plate. Launcher is shown closer to perch surface than in the real experiment for clarity. **b** Photograph of the soft robotic lander on the experiment landing surface. **c** Illustration showing the tendon driven tail’s operation with a servo-tensioning tendon to deflect the tail. **d** Image of the soft robotic lander perched on a tree outdoors, showing large pitch-back. **e**–**i** Robot model landing sequence, showing the pitch-back behavior and pose estimation from software (DeepLabCut). **j** Tracking result, showing the head, hip, and tail tip paths for successful (*n* = 22) and unsuccessful (*n* = 16) landings for a passive tail. **k** Ensemble mean of 22 successful landings out of 38 trials, showing the spread of pitch-back behavior (*n* = 22).

We conducted 79 landing attempts with the physical model orientated as observed in geckos from nature (Fig. 5a–d). The results showed quantitatively and qualitatively similar behavior between the robot model, our dynamic mathematical model, and the geckos (Fig. 5e–i), including an over-rotation after head and forefoot contact, followed by a pitch-back of the torso arrested by the tail with eventual recovery (Supplementary Movie 2). Looking in detail at the mechanics of the landing impact using pose measurements from the video shows why the tail reflex is advantageous. When the forefeet first contact the wall, the robot model’s translational momentum is quickly converted into angular momentum. This means that when the robot model’s hindfeet reach the landing surface, the model will tend to continue to rotate, and the front feet will detach if the adhesion force is insufficient. The robot model will then rotate away from the landing surface toward the ground as far as its geometry will permit. At this stage, a tailless robot is likely to detach its hindfeet from the wall, whereas a passive tail provides resistance to the pitch-back, and effectively acts as a rotary shock absorber.

A summary of the kinematics observed in videography analysis of the robot is plotted in Fig. 5j showing separately repeated successful and unsuccessful landings with a passive tail. As suggested by the dynamic model in the preceding section, the typical mode of failure for a landing is an uncontrolled pitch-back which results in loss of hindfeet contact, whereas successful landings arrest and recover the pitch-back. The pitch-back allows the robot model to dissipate the angular momentum it gained in the initial phase of the landing more slowly and with less force, and hence a more gradual complete deceleration and absorption of gliding kinetic energy than would be possible without pitch-back and partial detachment from the landing surface.

An ensemble average of 26 trials with a successful pitch-back recovery and an active tail reflex are shown in Fig. 5k, with the range of pitch-back and ensemble standard error plotted. The robot model showed a mean initial pitch-back rate of 1438°/s, compared to 2057°/s in the gecko observations. Similar peak pitch-back angles were found, but a slower recovery rate from that peak pitch-back angle, taking approximately three times as long to recover as it does to reach a maximum angle, unlike the more symmetric FAR observed in the geckos. This is primarily attributed to the passive silicone elastomer (Sil-30, printer Carbon Inc. M2) torso flexure, which is compliant and lacks the musculature of the animal. This attribution is supported by the theoretical pitch-back profiles (Fig. 4e), which do not account for any dissipative forces and are symmetric, unlike the robot and animal tests. Non-dimensionalizing the landing kinematics as $v/{L}_{B}{\dot{\theta }}_{0}$ (where $v$ is impact velocity, ${L}_{B}$ is body length, and ${\dot{\theta }}_{0}$ is the initial pitch-back angular velocity), which represents the linear kinetic energy of the impact relative to the rotational kinetic energy of the pitch-back, we get a mean value of 1.1 for the robot, and 4.0 for the gecko. The robot’s lower value indicates that it absorbs less energy in the initial phase of the impact and pitches back more rapidly relative to its impact velocity, likely a consequence of the use of material with reduced capacity to absorb energy compared to the active musculature of the gecko. Dynamic similarity of the pitch-back can be examined by calculating a dimensionless measure of the ratio of pitch-back centripetal force to gravitational acceleration, given by ${L}_{B}{\dot{\theta }}_{0}^{2}/g$ (where $g$ is acceleration due to gravity). This has a value of 2.7 × 10^4^ in the robot, and 1.9 × 10^4^ in the animal (see Supplementary Table S2), i.e., the weight force and centripetal force contributions are in similar proportion in the robot and in geckos.

### Physical model force measurements

**Fig. 6:**
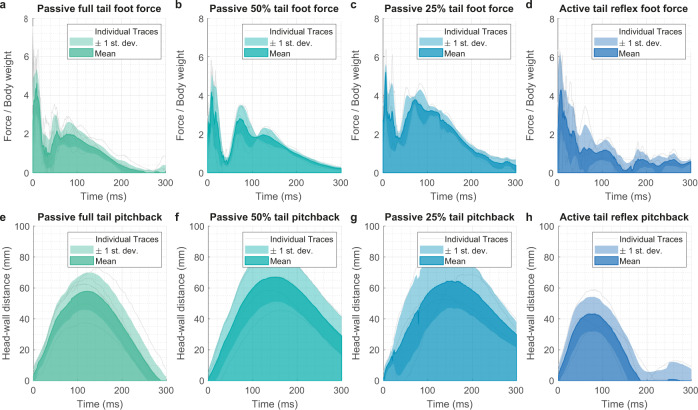
Wall-reaction foot force during pitch-back of fall-arresting response. (Supplementary Movie 3). **a**–**c** Ensemble average of foot force profiles during landing and pitch-back with varying lengths of a passive tail (*n* = 7, 5, and 4 respectively for **a**–**c**). Foot force is normalized by robot weight, and positive force represents adhesion with the landing plate. **d** Foot force with a full-length tail and an active tail reflex, triggered by forefoot contact (*n* = 5). Pitch-back angle is suppressed, and the force reduced from the passive case. **e**–**h** Distance between the robot model’s head and the landing plate over time, showing the stage of pitch-back in relation to each force profile (*n* = 7, 5, 4, and 5 respectively, showing the same trials as **a**–**d**).

We tested the robot model using a force sensor to record foot forces during landing (see Methods). In this experiment, the robot model was launched at a fixed 45° angle (reflecting the observed animal data in Fig. 1b, c), and a fixed launcher power setting. We tested the passive role of tail length and the effect of an active tail reflex, initiated by a contact switch. In each case, the average force over several trials was taken and an ensemble mean plotted (Supplementary Movie 3). These data are plotted in Fig. 6 with force plotted above the corresponding pitch-back angle. Results illustrated in Fig. 6a–c show that the shortened tail increased the force at the feet considerably, consistent with the mathematical model prediction and the observed difficulty tailless geckos have in landing. Across all three tail lengths, we see consistent pitch-back profiles indicating that the force increase is due in principal to the tail length change itself, and not to an effect of the tail loss on the torso dynamics.

In Fig. 6d, h, we see the effect of an active tail response with a full-length tail in which the detected contact of the forefeet with the wall initiated a downward curling of the tail, driven by a servo. We found that in these tests the tail action resulted in a steeper decrease in foot force than in the passive, full-length tail case, and a partial suppression of the pitch-back.

**Fig. 7:**
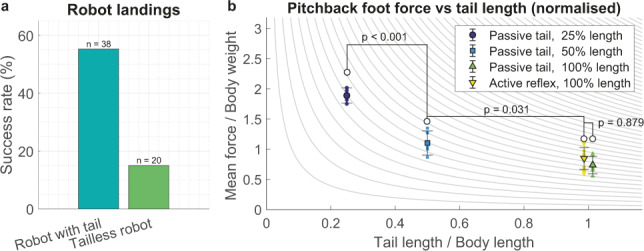
Robotic model data testing the role of tails in landing. **a** Landing success of the robotic model showed that tails substantially increased landing performance compared to tailless condition. **b** Plot of mean foot adhesive force during pitch-back for three different tested tail lengths (the mean of the force data plotted in Fig. 6a–d), and with an active tail reflex included. Isolines of force proportional to tail length^−1^ are plotted in the background for comparison with the relationship indicated by the theoretical model. Lines connecting bars indicate *P* values from ANOVA comparisons. The tail length datapoints for 100% tail length are offset ±1% to facilitate comparison between active and passive tails, though the true tail lengths are identical. Sample size, *n* = 4, 5, 7, and 5 for 25%, 50% and 100% passive and active tails, respectively.

Across the range of approach angles and velocities tested, we show that the tailed robot model outperformed the tailless platform, in a manner consistent with the observations of geckos in nature. The tailless robot model was able to land successfully in only 15% of trials (*n* = 20) compared to 55% with the tail present (*n* = 38) (Fig. 7a). The difference between tailed and tailless performance is significant (*χ*^2^ = 8.75, *P* = 0.0031, 1 df). If we then calculate mean force values on the feet for the experiments in Fig. 6, we find that the tail acts to reduce the force during deceleration (Fig. 7b) in a manner consistent with the simplified mechanical mathematical model we proposed, such that foot force increases in inverse proportion to tail length. We find statistically significant differences between different tail lengths (*F*(3,17) = 43, *P* < 10^−7^, analysis of variance [ANOVA]).

Results suggest that an active tail response can control landing as well, and reduce the instantaneous foot forces even further while reducing pitch-back during the response (Fig. 6d–h), although the additional force applied by tail actuation means the adhesion force does not go to zero (Fig. 6d), which lessens the reduction in mean force over the entire FAR (Fig. 7b). Our robotic physical model experiments (Figs 5 and 6) demonstrated the feasibility of landing stability enhanced by mechanical mediation using a tail as observed in unspecialized arboreal lizards and legged robots. We propose that such tail-type structures could potentially complement the predominantly aerodynamically controlled landings seen in birds^24^, as well as gliding robot planes^16^ to enhance robustness.

### The role of mechanical mediation in landing control of animals and robots

Results from the kinematics analysis of data collected in the field (Fig. 1) support the predictions from our hypothesized dynamic mathematical model (Fig. 2, Eqs (1)–(4)), suggesting that tail-type structures provide stability and could be important to mitigate hard landings. By contrast, gliding mammals (e.g., flying squirrels^37^) avoid hard landings and short glides since the steeper approach angles inhibit their ability to employ air braking by pitching up and to dissipate the high-impact energy across all four limbs simultaneously^38^.

Geckos possess both claws and adhesive pads, and both could contribute to attachment during impact. While it was not practical to measure the relative contributions of claws and setae in the gecko’s perching in this field experiment in the rainforest, previous work on geckos clinging to sawn wood showed that claw removal reduces attachment force^39^; however, the effect on macro-rough surfaces such as tree bark is not known. Work on invertebrates (Coleoptera beetles) has shown that claws do act synergistically with adhesive pads, but that the impact of claws is greatly reduced as the roughness size increases^40^. Even if adhesive and claw action can act synergistically to aid in adhesion in geckos, it is not clear if both are used in the FAR. The backwards rotation of the body and foot could make engagement of a curved claw more difficult to maintain relative to the adhesive pads. The relative importance of each of these adhesive systems during the FAR merits future investigation.

Strategies incorporating tail-assisted responses can increase the success of vertical landing performance and stability of both animals and robots. Asian flat-tailed geckos do not use a highly controlled stall with a substantial deceleration for landing during short-ranged glides. Small size permits a simple mechanically mediated solution for landing—a head-on collision where kinetic energy is absorbed^34^. Numerous small flying, running, and jumping animals such as bees^41^, fruit flies^42^, locusts^43^, and cockroaches^34,44^ undergo frequent collisions and head-on crash landings. Considering the effect of size, a small lizard of ~2 g might be able to crash-land into a tree without injury, whereas a ~2 kg flying lemur might experience injury and plastic deformation in the form of damage to tissues. Our hypothesis that tails increase landing stability by acting as a counter lever to the gecko’s body weight and thereby reduce foot forces is supported by both our theoretical and physical model, as well as other robotic perchers^15^. Animals and robots can use such mechanically mediated solutions to make landing control simpler. Tail responses during the landing behavior of geckos could be initiated by the same reflex discovered during climbing where forefoot slippage stimulates ventral flexion of the tail to provide support^4^. Capabilities associated with mid-air directed descent and landing in subcritical condition emerging in a relatively unspecialized arboreal lizard may support the notion that the emergence of behavior “precedes external morphological evolution”^45^.

For the design of robust multi-modal robots, we can learn strategies from nature that may lead to sufficient solutions for the problem of vertical landing in the face of a temporary loss of aerodynamic control or when challenged by unstructured terrains. Our study can provide inspiration for perching robots^19^ leading to greater landing stability and robustness to perturbations by using mechanically mediated designs to simplify control. We add one more unexpected function to the list of behaviors for gecko tails which support the assertion of Roderick et al.^25^ that, “the diversity in effective biological solutions offers ‘out of the box’ design inspiration for roboticists,” in this case, as it relates to landing strategies.

## Methods

### Animals

Our model system, the Asian flat-tailed gecko, *Hemidactylus platyurus*, is native to lowland tropical rainforest of Southeast Asia including wildlife reserves under the auspices of The Republic of Singapore (e.g.,^46–48^). The National Parks Board of The Republic of Singapore approved all field research and animal experiments were approved under Protocol # AUP-2017-03-9711-1 from U.C. Berkeley. The Wildlife Reserves Singapore allowed us to capture Asian flat-tailed geckos, *H. platyurus*, with Specimen Collection Permit # NP/RP955A. All methods of capture and handling are well-established standard techniques used by herpetologists. During collection, no harm was done to the animals or surrounding environment. All animals were released shortly after capture if they were deemed not suitable for the study. No detrimental effects resulted from the short confinement. Our study of perching behavior was based on 37 trials with 30 wild-caught *Hemidactylus platyurus* individuals. The average body weight of both tailed and tailless lizards was 2.2 ± 0.3 g, the average snout-vent length was 45 ± 2 mm, and the average tail length was 41 ± 3 mm (all measurements mean ±1 s.e.). According to the collection permit, wild-caught geckos were released after the conclusion of the locomotion experiments. Most lizards were released after a period of just 2 days in captivity. We temporarily accommodated lizards in portable terraria with ambient humidity (69.4 ± 5.3% s.e.) and temperature (33.0 ± 0.9 °C s.e.) for the shortest duration possible at our field site in the Wildlife Reserves. Water and crickets were provided ad libitum to animals during captivity.

### Field experiment protocol and measurements

We used generators to power two digital video cameras (X-PRI, AOS Technologies). To prevent damage of electronic equipment at humidity of ca. 69% and prevent overheating at temperatures of up to ~38 °C, we mounted heat sinks on the high-speed video cameras. Two cameras were placed on the ground orthogonal to the glide trajectory, with the long range camera 15.97 m away from glide plane with a 50 mm objective, and the close-range camera approximately 3.96 m away from the drop test zone using a 25 mm objective. Lighting conditions in the forest often changed rapidly, forcing us to adapt recording at different frame rates ranging from 120 to 500 frames per second. Lens apertures were also adjusted to ensure an adequate amount of light be made available to the high-speed video cameras.

Lizards were placed on a vertical platform at a height of 6.60 m from which they voluntarily took off. The majority of lizards that jumped off moved toward a tree trunk that was situated at a horizontal distance of 3.79 m away from the origin. Of the lizards that did not reach the tree trunk (*n* = 8), but landed short of it, most were seen to walk up to the tree trunk from the forest floor. This observation was encouraging in that the tree trunk proved to be a successful stimulus in triggering not only aerial, but also subsequent terrestrial locomotor behavior.

Prior to the landing experiments the wild-caught geckos were weighed and photographed for body length measurements. During these measurements the condition of their feet was evaluated and animals that were shedding were excluded from consideration for landing on a vertical target. We performed motion tracking using DLTdv5^49^ in MATLAB. We performed kinematic analysis and statistics using MATLAB. The gecko’s glide angle, velocities, and pitch angle were measured. Velocity and angle time series are filtered using a Savitzky–Golay filter with a 30-point span. The geckos’ pitch angle during gliding was measured relative to the horizontal. The duration for geckos to complete the FAR was measured at the onset of pitch-back of the torso away from the tree toward the forest floor until it recovered and returned the body to the tree trunk such that forefeet could establish contact with the tree (Fig. 2). The geckos’ pitch-back angle throughout the FAR was measured relative to the tree trunk. The duration that geckos remained at peak pitch-back angle (apex, Fig. 2h) was measured as the time window in which the instantaneous angular position of the body relative to the tree did not deviate from the peak angle by more than 2° that was considered to be within measurement error.

### Dynamic model

By resolving forces about the pivot point (Fig. 3), such that the centripetal acceleration and foot force, ${F}_{F}$, have no component, the rotational dynamics can be written as follows:

1 $$\left({I}_{B}+\frac{{m}_{B}{L}_{B}^{2}}{4}\right)\ddot{\theta }=\frac{{m}_{B}g{L}_{B}{\sin }\,\theta \,}{2}-{F}_{T}{L}_{T}$$

where $g$, ${m}_{B}$, and ${I}_{B}$ are gravitational acceleration, body mass, and rotational inertia, respectively. $\theta$ and $\ddot{\theta }$ are pitch-back angle and its second time derivative, angular velocity. Similarly, the normal force at the foot can be expressed by resolving forces in the horizontal direction:

2 $${F}_{T}+{F}_{F}=\frac{{m}_{B}{L}_{B}}{2}\left({\dot{\theta }}^{2}{\sin }\theta -\ddot{\theta }{\cos }\theta \right)\,$$

Combining Eqs (1) and (2) gives an expression for the normal force at the foot:

3 $${F}_{F}=\frac{{m}_{B}{L}_{B}}{2}\left({\dot{\theta }}^{2}{\sin }\theta -\ddot{\theta }{\cos }\theta \right)-\frac{1}{{L}_{T}}\left\{\,\frac{{m}_{B}g{L}_{B}{\cos }\theta \,}{2}-\left({I}_{B}+\frac{{m}_{B}{L}_{B}^{2}}{4}\right)\ddot{\theta }\right\}$$

The first linear dynamics term in Eq. (3) represents centripetal and angular acceleration forces. When the gecko’s body angular position is in the region of the average peak near orthogonal relative to the tree trunk, the component ${\cos }\theta$ goes to zero, as does angular velocity. Kinematics results (Fig. 2h) showed that geckos remained stationary for a brief period (mean 19 ± 3 ms, *n* = 3) at peak pitch-back angles. If body angular velocity goes to zero at an angle of approximately 90°, then the foot force equation becomes

as $$\dot{\theta }\to 0$$ and $$\theta \to \frac{\pi }{2}$$:

4 $${F}_{F}\to -\frac{1}{{L}_{T}}\left\{\,\frac{{m}_{B}g{L}_{B}}{2}-\left({I}_{B}+\frac{{m}_{B}{L}_{B}^{2}}{4}\right)\ddot{\theta }\right\}$$

Forces experienced at the foot, ${F}_{F}$, are thus inversely proportional to tail length.

The equation of motion (Eq. (1)) was numerically integrated in MATLAB, using an explicit Runge–Kutta solver (ode45) for each of the cases specified in the text. The initial angular velocity used as a boundary condition was taken as the mean gradient of the first five points of animal data shown in Fig. 4d.

### Perching robotic physical model

To investigate the landing mechanics of geckos in greater detail, we fabricated a simple robotic analog, with a soft body structure and an active tail reflex. The soft structures were rapidly prototyped in a silicone elastomer (Sil-30, Carbon Inc.) using a Carbon M2 printer and rigid components were produced in Nylon using a Markforged Mark Two. The soft tail is tendon driven (Fig. 5c) using 0.5 mm spring steel wire and actuated by a miniature servo (Blue Bird AMS102) permitting the testing of the tail reflex observed in the Asian flat-tailed gecko. Tail actuation input was provided by a microswitch on the robot’s underside and controlled with an on board SAMD21 microprocessor (Adafruit Trinket M0). The switch detected the point at which the underside of the head contacted the wall, and was programmed to actuate the tail whenever any detachment was detected with the microswitch. An RGB led was used to communicate the program state in video recordings, and a 100 mAh, 7.4 V lithium cell provides power. The total robot mass was 34.1 g, and a maximum length of 254 mm. The physical model was initially sized based on the observed scaling relationship between mass and snout-vent length in the infraorder Gekkota (i.e., mass = 10^−4.495^ × length^2.900^, with mass in g and length in mm, from^50^). Tail length was made equal to snout-vent length, as is typical in *H. platyurus* specimens.

For attachment to the wall, the robot has four compliant feet, each with a small Velcro pad. The compliant feet are made from TPU elastomer with a flexible geometry that allows them to translate and rotate in the robot model’s sagittal plane. The compliant feet aid attachment by providing some shock absorption and tolerance to misaligned impact, reflecting the action of the gecko’s limbs during a landing. The speed of the robot’s impact with the landing pad was increased to the point at which the feet’s attachment system began to fail, at 3–5 m/s, such that tail’s role was critical.

### Experimental setup

The catapult used stretched elastic (8 mm diameter, 1-mm thick latex tubes) to propel a carriage along a linear guide rail with recirculating ball bearings toward a rubber stopper. The carriage was pulled backward, tensioning the elastic, and released, with the release point adjustable in 20 mm increments. The robot model was held on to the carriage by an electromagnet prior to and during launch. The electromagnet was deactivated by a trigger switch 1 cm from the end of the rail, releasing the robot without any perturbation from the carriage’s impact with the stopper (Fig. 5a and Supplementary Movie 2). The rig can be repositioned to change approach angle, and a digital inclinometer was attached to the linear rail to ensure orientation accuracy to within 0.1°. The aerial phase between launch and landing resulted in variation in the angle and speed of the robot at impact. This is plotted in Supplementary Fig. S1.

The landing surface was an 8-mm thick wooden plate covered by a felt fabric sheet (see Supplementary Fig. S1 for felt fabric attachment forces). For force measurement, this plate was placed above a section of separated plate connected to a force sensor, such that only the robotic model’s hindfoot made contact with the instrumented plate section during landing covered with velcro (see the “Landing substrate” section in Supplementary Material file), while the tail and forefeet contact landing plates covered in felt are mounted directly to the wall via vibration isolators to reduce sensor noise. The 6-axis force sensor (ATI Nano17 titanium) recorded the forces generated in the foot as the robot model impacted the wall at 10 kHz, while a videography camera (AOS S-motion) recorded the motion at 800 fps (Fig. 5e–i).

Pose information was extracted from video data using the open source DeepLabCut^51^ library, which offers markerless tracking using deep learning. High-contrast features on the robot model provided tracking fiducials and allowed recording of 2D pose and velocity. The network was trained for 400,000 iterations with a training set of 100 images. Pixel lengths were calibrated against the dimensions of the robot model itself for each individual video, and the camera was leveled with an inclinometer to align the image coordinate system with the vertical wall. All post processing was done in MATLAB.

### Statistics and reproducibility

All statistical tests were performed in MATLAB. Two sets of physical model experiments were conducted. Landing success for tailed and tailless robot trials was compared using a *χ*^2^ test for independence. The relationship between tail length and landing force was also examined using a one-way ANOVA. The criterion for statistical significance was *P* < 0.05 for all tests.

### Reporting summary

Further information on research design is available in the Nature Research Reporting Summary linked to this article.

## Supplementary information

Supplementary Material

Description of Additional Supplementary Files

Movie 1. Field footage of gliding geckos landing on a tree.

Movie 2. Soft robotic physical model of the gecko landing.

Movie 3. Force measurements of the soft robotic physical model of the gecko landing.

Reporting Summary

## Data Availability

A repository has been created with kinematics tracking data for all glides (.csv files), hosted on an open research data repository in 10.17617/3.6d^52^.

## Code availability

The data repository also includes the MATLAB processing script used to analyze and plot the gecko tracking data (hosted on an open research data repository: 10.17617/3.6d^52^).

## References

[1] Autumn K (2000). Evidence for van der Waals adhesion in gecko setae. Proc. Natl Acad. Sci. USA..

[2] Autumn K, Dittmore A, Santos D, Spenko M, Cutkosky M (2006). Frictional adhesion: a new angle on gecko attachment. J. Exp. Biol..

[3] Autumn K (2000). Adhesive force of a single gecko foot-hair. Nature.

[4] Jusufi A, Goldman DI, Revzen S, Full RJ (2008). Active tails enhance arboreal acrobatics in geckos. Proc. Natl Acad. Sci. USA..

[5] Mongeau JM (2012). Rapid inversion: running animals and robots swing like a pendulum under ledges. PLoS One.

[6] Nirody JA (2018). Geckos race across the water’s surface using multiple mechanisms. Curr. Biol..

[7] Autumn K (2006). Dynamics of geckos running vertically. J. Exp. Biol..

[8] Honda M (1997). Cosymbotus craspedotus (Frilly Gecko) and C. platyurus (Flat-tailed Gecko) gliding behavior. Herpetol. Rev..

[9] Heinicke MP, Greenbaum E, Jackman TR, Bauer AM (2012). Evolution of gliding in Southeast Asian geckos and other vertebrates is temporally congruent with dipterocarp forest development. Biol. Lett..

[10] Jusufi, A., Kawano, D. T., Libby, T. & Full, R. J. Righting and turning in mid-air using appendage inertia: reptile tails, analytical models and bio-inspired robots. *Bioinspir. Biomim.* **5**, 045001 (2010). 10.1088/1748-3182/5/4/045001 21098954

[11] Khandelwal PC, Hedrick TL (2020). How biomechanics, path planning and sensing enable gliding flight in a natural environment. Proc. R. Soc. B..

[12] Willis, D., Bahlman, J., Breuer, K. S. & Swartz, S. Energetically optimal short-range gliding trajectories for gliding animals. *AIAA J*. **49** (2011).

[13] Chapple, D. G. & Swain, R. Effect of caudal autotomy on locomotor performance in a viviparous skink, *Niveoscincus metallicus*. *Funct. Ecol*. **16**, 817–825 (2002).

[14] Gravish N, Lauder GV (2018). Robotics-inspired biology. J. Exp. Biol..

[15] Estrada, M. A., Hawkes, E. W., Christensen, D. L. & Cutkosky, M. R. Perching and vertical climbing: design of a multimodal robot. In *2014 IEEE International Conference on Robotics and Automation (ICRA)* 4215–4221 (2014). 10.1109/ICRA.2014.6907472.

[16] Lussier Desbiens, A., Asbeck, A. T. & Cutkosky, M. R. Landing, perching and taking off from vertical surfaces. *Int. J. Robot. Res.* **30**, 355–370 (2011).

[17] Kovac, M., Germann, J. & Hürzeler, C. A perching mechanism for micro aerial vehicles. *J. Micro-Nano Mech*. 77–91 (2009). 10.1007/s12213-010-0026-1.

[18] Pope MT (2017). A multimodal robot for perching and climbing on vertical outdoor surfaces. IEEE Trans. Robot..

[19] Kovac M (2016). Learning from nature how to land aerial robots. Science.

[20] Dudley R (2007). Gliding and the functional origins of flight: biomechanical novelty or necessity?. Annu. Rev. Ecol. Evol. Syst..

[21] Dudley, R. & Yanoviak, S. P. Animal aloft: the origins of aerial behavior and flight. *Integr. Comp. Biol.* **51**, 926–936 (2011). 10.1093/icb/icr002. 10.1093/icb/icr002 21558180

[22] Socha JJ, Jafari F, Munk Y, Byrnes G (2015). How animals glide: from trajectory to morphology. Can. J. Zool..

[23] Bonser RHC (1999). Branching out in locomotion: the mechanics of perch use in birds and primates. J. Exp. Biol..

[24] Provini P, Tobalske BW, Crandell KE, Abourachid A (2014). Transition from wing to leg forces during landing in birds. J. Exp. Biol..

[25] Roderick WR, Cutkosky MR, Lentink D (2017). Touchdown to take-off: at the interface of flight and surface locomotion. Interface Focus.

[26] McGuire JA, Dudley R (2005). The cost of living large: comparative gliding performance in flying lizards (Agamidae: Draco). Am. Nat..

[27] Byrnes G, Lim NTL, Spence AJ (2008). Take-off and landing kinetics of a free- ranging gliding mammal, the Malayan colugo (*Galeopterus variegatus*). Proc. R. Soc. B.

[28] Maximilian Dehling, J. How lizards fly: a novel type of wing in animals. *PLoS One* **13**, e0189573 (2017). 10.1371/journal.pone.0189573 PMC5728497 29236777

[29] Young. BA, LEE. CE, Daley. MK (2002). On a flap and a foot: aerial locomotion in the flying gecko, *Ptychozoon kuhli*. J. Herpetol..

[30] Marcellini DL, Keefer TE (1976). Analysis of the gliding behavior of *Ptychozoon lionatum* (Reptilia: Gekkonidae). Herpetologica.

[31] Vanhooydonck B (2009). Ecomorphological analysis of aerial performance in a non-specialized lacertid lizard, *Holaspis guentheri*. J. Exp. Biol..

[32] Graham, M. & Socha, J. J. Going the distance: the biomechanics of gap-crossing behaviors. *J. Exp. Zool. Part A Ecol. Integr. Physiol*. **333**, 60–73 (2020). 10.1002/jez.2266 31111626

[33] Wang, H., Wang, W., Song, Y., Cai, L. & Dai, Z. Passive cushiony biomechanics of head protection in falling geckos. *Appl. Bionics. Biomech*. **2018**, 9857894 (2018). 10.1155/2018/9857894 PMC5836436 29670666

[34] Jayaram, K. et al. Transition by head-on collision: mechanically mediated manoeuvres in cockroaches and small robots. *J. R. Soc. Interface* **15**, 20170664 (2018). 10.1098/rsif.2017.0664 PMC5832722 29445036

[35] Higham TE, Russell AP, Niklas KJ (2017). Leaping lizards landing on leaves: escape-induced jumps in the rainforest canopy challenge the adhesive limits of geckos. J. R. Soc. Interface.

[36] Gravish N (2009). Rate-dependent frictional adhesion in natural and synthetic gecko setae. J. R. Soc. interface.

[37] Ando M, Shiraishi S (1993). Gliding flight in the Japanese Giant Flying Squirrel Petaurista leucogenys. J. Mamm. Soc. Jpn..

[38] Paskins KE, Bowyer A, Megill WM, Scheibe JS (2007). Take-off and landing forces and the evolution of controlled gliding in northern flying squirrels *Glaucomys sabrinus*. J. Exp. Biol..

[39] Naylor, E. R. & Higham, T. E. Attachment beyond the adhesive system: the contribution of claws to gecko clinging and locomotion. *Integr. Comp. Biol*. **59**, 168–181 (2019). 10.1093/icb/icz027 31070737

[40] Song, Y., Dai, Z., Wang, Z., Ji, A. & Gorb, S. N. The synergy between the insect-inspired claws and adhesive pads increases the attachment ability on various rough surfaces. *Sci. Rep*. **6**, 26219 (2016). 10.1038/srep26219 PMC4873747 27198650

[41] Crall JD, Ravi S, Mountcastle AM, Combes SA (2015). Bumblebee flight performance in cluttered environments: effects of obstacle orientation, body size and acceleration. J. Exp. Biol..

[42] van Breugel F, Dickinson MH (2012). The visual control of landing and obstacle avoidance in the fruit fly Drosophila melanogaster. J. Exp. Biol..

[43] Reichel SV, Labisch S, Dirks J-H (2019). What goes up must come down: biomechanical impact analysis of falling locusts. J. Exp. Biol..

[44] Kesel AB, Martin A, Hoffmann F (2009). Quantifying the landing reaction of cockroaches. Final Rep. Ariadna Study.

[45] Wcislo WT (1989). Behavioral environments and evolutionary change. Annu. Rev. Ecol. Syst..

[46] Marcellini, D. & Keefer, T. Analysis of the gliding behavior of *Ptychozoon lionatum* (Reptilia: Gekkonidae). *Herpetologica* **32**, 362–366 (1976).

[47] Chou LM (1978). Some bionomic data on the house geckos of Singapore. Malay. Nat. J..

[48] Corlett R (1997). The vegetation in the nature reserves of Singapore. Gard. Bull. Singap..

[49] Hedrick TL (2008). Software techniques for two- and three-dimensional kinematic measurements of biological and biomimetic systems. Bioinspiration Biomim..

[50] Meiri S (2010). Length-weight allometries in lizards. J. Zool..

[51] Mathis A (2018). DeepLabCut: markerless pose estimation of user-defined body parts with deep learning. Nat. Neurosci..

[52] Jusufi, A., Siddall, R., Full, R., & Byrnes, G. Tails stabilize landing of gliding geckos crashing head-first into tree trunks. Max Planck Society. 10.17617/3.6d (2021). 10.1038/s42003-021-02378-6 PMC8413312 34475510

