# Supplementary material for: Tails stabilize landing of gliding geckos crashing head-first into tree trunks
Source: Commun Biol. 2021 Sep 2;4:1020. doi: 10.1038/s42003-021-02378-6 (PMC8413312; doi:10.1038/s42003-021-02378-6)
Supplement: Supplementary file 1 — Supplementary Material [file 42003_2021_2378_MOESM1_ESM.pdf]

Tails stabilize landing of gliding geckos crashing head-first into tree trunks

Robert Siddall[1], Greg Byrnes[2], Robert J. Full[3] & Ardian Jusufi[*1,3]

[1]Locomotion in Biorobotic and Somatic Systems, Max Planck Institute for Intelligent Systems, Stuttgart, Germany.

[2]Department of Biology, Siena College, Loudonville, USA.

[3]Department of Integrative Biology, University of California at Berkeley, Berkeley, USA.

Supplementary table 1: Summary of morphology from the animal experiments, including number of trials recorded. All measurements mean ± s.e.

Individuals	Trials	SVL (mm)	Tail (mm)	Mass (g)
30	*37*	*45 ± 2*	*41 ± 3*	*2.2 ± 0.3*

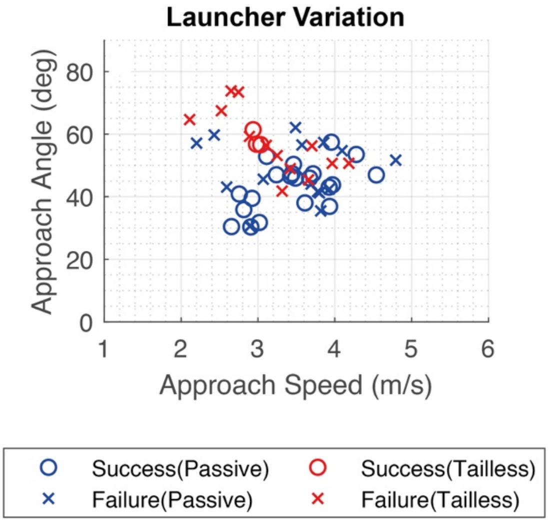

Supplementary figure S1: Variation of landing speeds and angles for robot model. The aerial phase between launch and landing results in some variation from the fixed launch speeds and angles. This graph shows the condition of the robot model at impact for various test configurations.

Supplementary table 2: Comparison of robot and animal morphology and pitchback kinematics, showing masses, m_B, body length, L_B, impact velocity, v, initial pitchback angular velocity, $\dot{\theta}_0$, and foot force, F_F.

Parameter	Unit	Gecko	Robot
m_B	*kg*	0.0023	0.0341
L_B	*m*	0.045	0.127
v	*m/s*	6.0	3.6
F_F	*N*	-	0.23
$\dot{\theta}_0$	*deg/s*	2057	1438

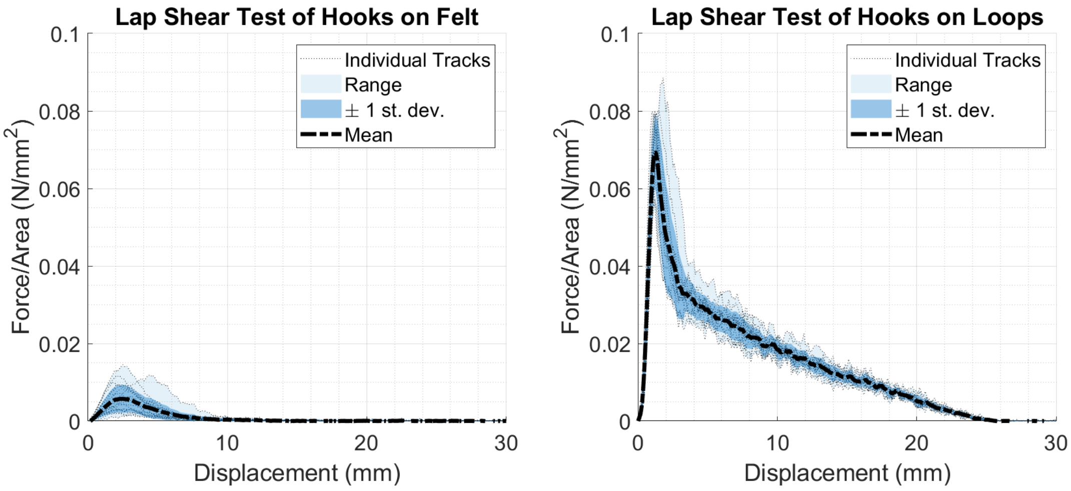

Supplementary Figure S2: Performance of robot feet on landing substrate. The robot's feet are formed by the 'hook' portion of a hook and loop fastener (brand: RS-Pro), while the landing substrate is made of 3mm thick soundproofing felt (brand: RS-Pro). Instron tests of a 15 x 45 mm sample (15 repetitions) show that replacing the 'loop' portion of the fastener with the felt reduces peak force by a factor of 11 and reduces work done in detachment by a factor of 514, compared to the paired hook-and-loop fastener.

Landing Substrate

During the tests measuring landing success rate (Fig. 5, Supplementary figure S2, Supplementary movie 2), the entire landing surface was covered in felt. For tests measuring pitchback forces, the landing surface was also covered in felt, but a section of 'loop' Velcro fastener was used to cover the force plate (see Supplementary movie 3). This allowed us to measure large attachment forces at the hindfeet that would have otherwise caused the hindfeet to detach, particularly where the robot tail was shortened and forces increased (Fig. 7). Loops/Velcro were only used at the force plate, the remainder of the landing surface was covered in felt, such that the tail and fore feet were on felt, not loops, the same as the landing success rate tests.
