# Supplementary material for: Tails stabilize landing of gliding geckos crashing head-first into tree trunks
Source: Commun Biol. 2021 Sep 2;4:1020. doi: 10.1038/s42003-021-02378-6 (PMC8413312; doi:10.1038/s42003-021-02378-6)
Supplement: Supplementary file 2 — Description of Additional Supplementary Files [file 42003_2021_2378_MOESM2_ESM.pdf]

Description of Additional Supplementary Files

File Name: Supplementary Movie 1
Description: A gecko, *Hemidactylus platyurus,* in the Southeast Asian lowland tropical rainforest. The lizard releases from the underside of a vertically mounted platform 6.6 meters above ground. After the initial free falling or jump phase, the animal goes into a non-equilibrium glide and crashed head-first, reaching the tree stimulus after covering 4.3m horizontal transit. Movie was recorded in the rainforest at 250 frames/second, and is replayed slowed x2 and slowed x10. The gecko's position is highlighted with a green circle. **Sequence 2.** Perspective is on *Hemidactylus platyurus* in one quadrant of the glide arena between mid-glide and landing. In the close-up views, we measured changes in body attitude over time and observed the Fall-Arresting Response (FAR) showing substantial body pitch-back and recovery back to the tree trunk. Footage was originally recorded in the rainforest at 500 frames/second. The gecko's position is highlighted with a green circle. **Sequence 3.** Three views of the same *Hemidactylus platyurus* individual without a tail failing to alight on the tree. Footage was originally recorded in the rainforest at 500 frames/second, and is replayed slowed x2 and slowed x10. The gecko's position is highlighted with a green circle in two views.

File Name: Supplementary Movie 2
Description: Montage video showing soft robotic physical model used in experiments. Sequence shows the robot lander compared with the gecko landing, followed by the active tail reflex, including the contact response, manually triggered, and the launch system used to recreate landings. **Sequence 2.** Examples of a landing attempt with a passive tail, without a tail, and with an active tail, showing the inability of the robot to maintain contact with the wall without a tail. Video recording and display speeds are indicated in the video subtitles.

File Name: Supplementary Movie 3
Description: Examples of a landing attempt with an active tail and a short, passive tail, with average force over repeated trials shown in an animated plot. The movie shows the reduction in adhesion requirements from a shorter tail. Video recording and display speeds are indicated in the video subtitles.
