# Supplementary material for: Tails stabilize landing of gliding geckos crashing head-first into tree trunks
Source: Commun Biol. 2021 Sep 2;4:1020. doi: 10.1038/s42003-021-02378-6 (PMC8413312; doi:10.1038/s42003-021-02378-6)
Supplement: Supplementary file 6 — Reporting Summary [file 42003_2021_2378_MOESM6_ESM.pdf]

nature research

Corresponding author(s): Ardian Jusufi

Last updated by author(s): Jun 2, 2021

Reporting Summary

Nature Research wishes to improve the reproducibility of the work that we publish. This form provides structure for consistency and transparency in reporting. For further information on Nature Research policies, see our Editorial Policies and the Editorial Policy Checklist.

Statistics

For all statistical analyses, confirm that the following items are present in the figure legend, table legend, main text, or Methods section.

n/a	Confirmed	
☐	☒	The exact sample size (*n*) for each experimental group/condition, given as a discrete number and unit of measurement
☐	☒	A statement on whether measurements were taken from distinct samples or whether the same sample was measured repeatedly
☐	☒	The statistical test(s) used AND whether they are one- or two-sided<br>*Only common tests should be described solely by name; describe more complex techniques in the Methods section.*
☐	☒	A description of all covariates tested
☐	☒	A description of any assumptions or corrections, such as tests of normality and adjustment for multiple comparisons
☐	☒	A full description of the statistical parameters including central tendency (e.g. means) or other basic estimates (e.g. regression coefficient) AND variation (e.g. standard deviation) or associated estimates of uncertainty (e.g. confidence intervals)
☐	☒	For null hypothesis testing, the test statistic (e.g. F, t, r) with confidence intervals, effect sizes, degrees of freedom and P value noted<br>*Give P values as exact values whenever suitable.*
☒	☐	For Bayesian analysis, information on the choice of priors and Markov chain Monte Carlo settings
☒	☐	For hierarchical and complex designs, identification of the appropriate level for tests and full reporting of outcomes
☒	☐	Estimates of effect sizes (e.g. Cohen's *d*, Pearson's *r*), indicating how they were calculated

Our web collection on statistics for biologists contains articles on many of the points above.

Software and code

Policy information about availability of computer code

Data collection	Software used: AOS Imaging Studio, LabView
Data analysis	Software used: Matlab (R2019b), DeepLabCut (opensource, latest version on 7/15/2019 used) and Microsoft Excel

For manuscripts utilizing custom algorithms or software that are central to the research but not yet described in published literature, software must be made available to editors and reviewers. We strongly encourage code deposition in a community repository (e.g. GitHub). See the Nature Research guidelines for submitting code & software for further information.

Data

Policy information about availability of data

All manuscripts must include a data availability statement. This statement should provide the following information, where applicable:

- Accession codes, unique identifiers, or web links for publicly available datasets
- A list of figures that have associated raw data
- A description of any restrictions on data availability

Data supporting the findings of this study are available from the corresponding author upon reasonable request.

Field-specific reporting

Life sciences study design

All studies must disclose on these points even when the disclosure is negative.

Sample size	Our study of perching behavior was based on 37 trials with 30 wild-caught Hemidactylus platyurus individuals.
Data exclusions	No data was excluded from the analyses.
Replication	No independent replication of this study was attempted.
Randomization	A single species was studied for the purpose of this study, and no experimental grouping or randomization was used, all animals suitable for the study were used in the experiment.
Blinding	The experiment involved no grouping or allocation, and so blinding was not required, all animals suitable for the study were used in the experiment.

Reporting for specific materials, systems and methods

We require information from authors about some types of materials, experimental systems and methods used in many studies. Here, indicate whether each material, system or method listed is relevant to your study. If you are not sure if a list item applies to your research, read the appropriate section before selecting a response.

Materials & experimental systems

n/a	Involved in the study	
☒	☐	Antibodies
☒	☐	Eukaryotic cell lines
☒	☐	Palaeontology and archaeology
☐	☒	Animals and other organisms
☒	☐	Human research participants
☒	☐	Clinical data
☒	☐	Dual use research of concern

Methods

n/a	Involved in the study	
☒	☐	ChIP-seq
☒	☐	Flow cytometry
☒	☐	MRI-based neuroimaging

Animals and other organisms

Policy information about studies involving animals; ARRIVE guidelines recommended for reporting animal research

Laboratory animals	The study did not involve laboratory animals.
Wild animals	The National Parks Board of The Republic of Singapore approved all field research and the Wildlife Reserves Singapore allowed us to capture Asian flat-tailed geckos, Hemidactylus platyurus, with Specimen Collection Permit # NP/RP955A. Experiments were also approved under Protocol # AUP-2017-03-9711-1 from U.C. Berkeley. When we collected lizards no harm was done to them or surrounding environment. All lizards were released shortly after capture if they were deemed not suitable for the study. No detrimental effects resulted from the short confinement. All methods of capture and handling are well-established standard techniques used by herpetologists. Our study of perching behavior was based on wild-caught H. platyurus. All lizards were released after the conclusion of the locomotion experiments. Most lizards were released after a period of just two days in captivity. We made sure that lizards were provided with water at all times. We fed crickets to the lizards which were provided by the veterinarians at the Animal Healthcare Hospital, who accompanied the project. We temporarily accommodated lizards in portable terraria with ambient humidity (ca. 85%) and temperature (ca. 38°C) for the shortest duration possible at our field site in the Wildlife Reserves.
Field-collected samples	The study did not involve field-collected samples.
Ethics oversight	The Wildlife Reserves Singapore provided oversight on the project. Guidance on animal husbandry was provided by veterinarians at the Animal Healthcare Hospital. The experimental protocol was approved by the U.C. Berkeley Animal Care and Use Committee.

Note that full information on the approval of the study protocol must also be provided in the manuscript.
